# Predicting subclinical leaflet thrombosis in self-expandable prosthesis: A multimodal machine learning analysis

**DOI:** 10.1016/j.xjse.2025.100064

**Published:** 2025-07-29

**Authors:** Marco Moscarelli, Thanos Athanasiou, Roberto Casula, Vincenzo Pernice, Gregorio Zaccone, Adriana Zlahoda-Huzior, Dariusz Dudek, Sabrina Milo, Giuseppe Speziale, Antonio Fabbrizio, Khalil Fattouch

**Affiliations:** a Department of Cardiovascular Surgery, Maria Eleonora Hospital, GVM Care&Research, Palermo, Italy; b Department of Surgery & Cancer, Imperial College London, Faculty of Medicine, London, United Kingdom; c Department of Cardiothoracic Surgery, Hammersmith Hospital, Imperial College Healthcare NHS Trust, London, United Kingdom; d Simhub, Virmed, Krakow, Poland; e Center for Digital Medicine and Robotics, Jagiellonian University Medical College, Krakow, Poland; f Department of Radiology, Maria Eleonora Hospital, GVM Care&Research, Palermo, Italy; g Department of Theoretical and Applied Sciences, eCampus University, Novedrate, Italy; h Kore University of Medicine, Enna, Italy

**Keywords:** machine learning, subclinical leaflet thrombosis, transcatheter aortic valve replacement

## Abstract

**Objective:**

Subclinical leaflet thrombosis is a known finding after transcatheter aortic valve implantation, but its predictors remain poorly defined. Machine learning offers new opportunities for identifying complex, nonlinear relationships among clinical, anatomic, and hematological variables.

**Methods:**

We analyzed data from 118 patients who underwent transcatheter aortic valve implantation with self-expanding valves and scheduled multidetector computed tomography at 6 months. A total of 120 preprocedural and postprocedural variables were included. Three machine learning models, least absolute shrinkage and selection operator logistic regression, Random Forest, and Extreme Gradient Boosting, were trained and internally validated using stratified 5-fold cross-validation.

**Results:**

Subclinical leaflet thrombosis was identified in 22 patients (18.6%). Bicuspid aortic valve morphology emerged as one of the strongest predictors across all machine learning models (least absolute shrinkage and selection operator β = 1.33; Gini = 1.31; SHapley Additive exPlanations = 0.42). Other top predictors included serum creatinine (β = 0.29; Gini = 0.90), hemoglobin decrease (β = 0.05; Gini = 1.32; SHapley Additive exPlanations = 0.10), hematocrit decrease (β = 0.02; Gini = 1.43; SHapley Additive exPlanations = 0.11), and platelet nadir (SHapley Additive exPlanations = 0.09). All models demonstrated strong discriminative ability (area under the curve range, 0.84-0.89; Brier scores: 0.040-0.163).

**Conclusions:**

This is the first study to apply a multimodal machine learning framework to predict subclinical leaflet thrombosis after transcatheter aortic valve implantation. Bicuspid anatomy and perioperative hematological changes were consistently associated with subclinical leaflet thrombosis, highlighting the potential of machine learning to enhance postprocedural risk stratification. Incorporating routinely available variables into machine learning models may help guide early imaging and personalized antithrombotic strategies.

## Graphical Abstract

**Figure:**
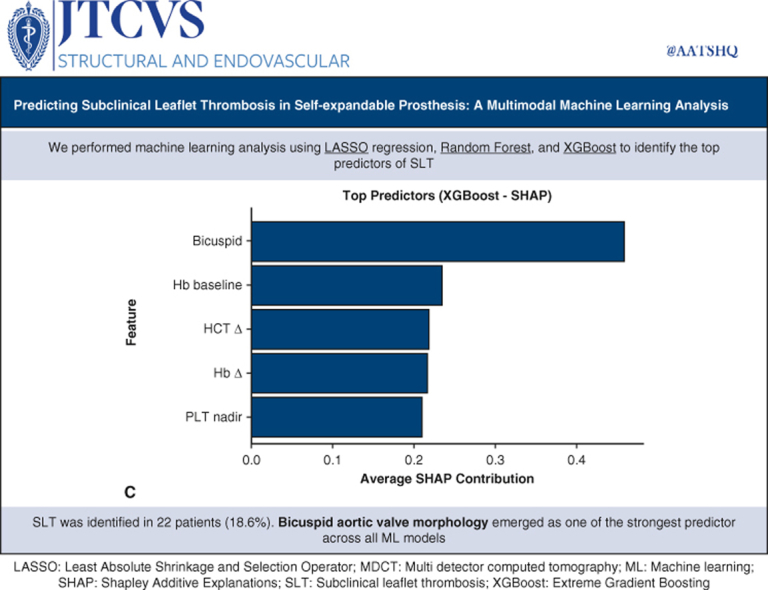

**Figure:**
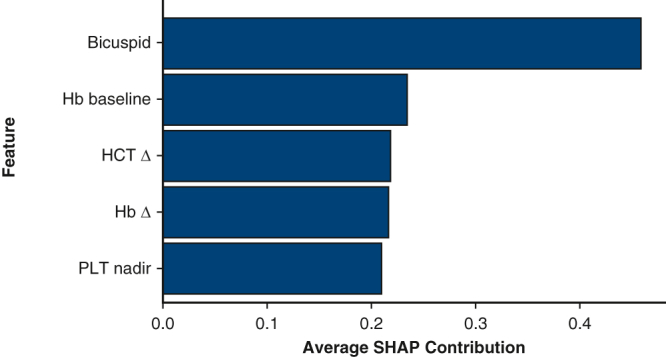
ML-derived predictor of subclinical leaflet thrombosis.

**Central Message**

ML analysis of multimodal data identifies bicuspid anatomy and early postoperative hematological shifts as key predictors of subclinical leaflet thrombosis after TAVI.

**Perspective**

Subclinical leaflet thrombosis after TAVI may be anticipated by routinely collected clinical information. In this study, bicuspid valve morphology and early postoperative changes in hemoglobin, hematocrit, and platelet counts were associated with increased SLT risk. Recognizing these signals may help clinicians tailor imaging surveillance and consider preventive treatment strategies in selected patients.

The indications for transcatheter aortic valve implantation (TAVI) have broadened considerably in recent years, with the procedure now established as a first-line therapy for low-risk patients in many clinical settings.^1^^,^^2^

TAVI is also being evaluated in asymptomatic individuals with severe aortic stenosis,^3^ and emerging data support its use in selected patients with moderate aortic stenosis and impaired left ventricular function.^4^ As the procedure is increasingly adopted in younger and lower-risk populations, attention has shifted toward long-term outcomes, particularly prosthesis durability and early signs of dysfunction.^5^

Among these, subclinical leaflet thrombosis (SLT), also known as “hypoattenuated leaflet thickening (HALT),” has emerged as a frequent finding on multidetector computed tomography (MDCT) and may be considered as an early manifestation of structural valve deterioration.^5^, ^6^, ^7^ Despite growing recognition of SLT, its underlying mechanisms and predictors remain incompletely defined.

SLT appears to be a multifactorial process, influenced by complex, nonlinear interactions among clinical, imaging, and hematological parameters.^5^^,^^7^^,^^8^ These patient–prosthesis host interactions are often challenging to characterize using conventional statistical approaches, especially in the context of limited sample sizes, incomplete longitudinal follow-up, and restricted availability of high-resolution imaging modalities such as MDCT.

To address this limitation, we used a multimodal machine learning (ML) approach, incorporating least absolute shrinkage and selection operator (LASSO) regression, Random Forest, and Extreme Gradient Boosting (XGBoost). These methods do not rely on strict statistical assumptions and are well suited to detect patterns and variable interactions that may not be fully captured by traditional models.^9^

## Material and Methods

The present study was part of the EndoTAVI Project, a European grant–funded study (P.O.C. FESR SICILIA 2014/2020, Azione 1.1.1/https://www.euroinfosicilia.it/poc-20142020-azione, other details at www.endotavi.it) to evaluate predictors of early dysfunction after TAVI, conducted at Maria Eleonora Hospital GVM Care & Research, a tertiary university center in Palermo, Italy.

This prospective study included 118 consecutive patients with severe aortic valve stenosis who underwent transfemoral TAVI with the Evolut R/PRO (Medtronic Inc) or Portico/Navitor (Abbott Structural Heart) between January 2019 and July 2024, and 2-dimensional transthoracic echocardiography and MDCT evaluation at 6-month follow-up. Patients were enrolled before the procedure. The full inclusion and exclusion criteria have been reported^10^ and are provided in the Appendix E1.

The study was performed in accordance with the Second Declaration of Helsinki, followed by the Strengthening the Reporting of Observational Studies in Epidemiology^11^ recommendations and was approved by the Scientific Ethics Committee of the University of Palermo (Comitato Etico - University of Palermo, N:1/2020, date of approval 06/01/2020). All patients provided written informed consent before enrollment in the study.

### Study Design and Dataset Preparation

This study aimed to identify predictors of SLT using a combination of LASSO regression, Random Forest, and XGBoost models.^12^, ^13^, ^14^ The dataset included a comprehensive set of clinical, laboratory, and imaging variables. Specifically, it comprised (1) demographic and (2) clinical characteristics; (3) pre- and postprocedural echocardiographic parameters; (4) procedural variables; (5) MDCT imaging at baseline and follow-up; and (6) longitudinal blood markers collected during the first week postimplantation and at 30 and 60 days (full dataset provided in Table E1).

To better capture dynamic trends, blood markers were also analyzed using derived metrics: delta (difference between baseline and nadir), mean (average across time points), and nadir (lowest recorded value).

### Patient Selection

Patients with severe symptomatic aortic stenosis who underwent TAVI with Evolut R/PRO, Portico, or Navitor valves were eligible for inclusion. Key exclusion criteria comprised aneurysm of the Valsalva or ascending aorta, previous aortic root replacement, pure aortic regurgitation, other significant valvular disease, moderate to severe renal impairment (eGFR <40 mL/min/1.73 m^2^), inability to achieve a heart rate less than 60 bpm during MDCT (eg, uncontrolled atrial fibrillation), allergy to iodine contrast, and refusal or inability to provide informed consent. Participation in another clinical study also constituted an exclusion. Notably, the use of dual antiplatelet therapy, vitamin K antagonists, or direct oral anticoagulants/novel oral anticoagulants did not preclude enrollment.

### Imaging Analysis

Subclinical leaflet thrombosis was adjudicated as previously described.^10^^,^^15^ Briefly, pre- and postprocedural contrast-enhanced MDCT scans were acquired using a Siemens SOMATOM Drive scanner (VB20, 2019). Image analysis was conducted with dedicated postprocessing software (Syngo.via, Siemens Healthcare GmbH). A full protocol of imaging acquisition is in the Appendix E1.

Advanced 3-dimensional multiparametric digital approach was also used to visualize valvular thrombosis and its spatial relationship with surrounding anatomic structures, as previously described.^16^

Leaflet thrombosis was identified as HALT/SLT, characterized by a meniscal-shaped thickening (>2 mm) on the prosthetic leaflet surface with an attenuation of less than 200 Hounsfield units.^7^ These lesions were detected in the diastolic phase of leaflet coaptation on at least 2 imaging planes. The extent of HALT was classified along the leaflet's long axis from the base using a 5-tier grading system. The per-patient severity was determined based on the leaflet with the highest HALT grade.^17^

Restricted leaflet movement (RELM) was defined as hypoattenuated thickening observed during systole, leading to impaired leaflet mobility in 1 or more leaflets. RELM severity was categorized into 5 groups: no RELM, RELM 25% or less, RELM more than 25% to 50%, RELM greater than 50% to 75%, and RELM greater than 75%.^17^ A HALT lesion associated with RELM greater than 50% was considered a threshold for significant leaflet motion restriction.

**Figure E1:**
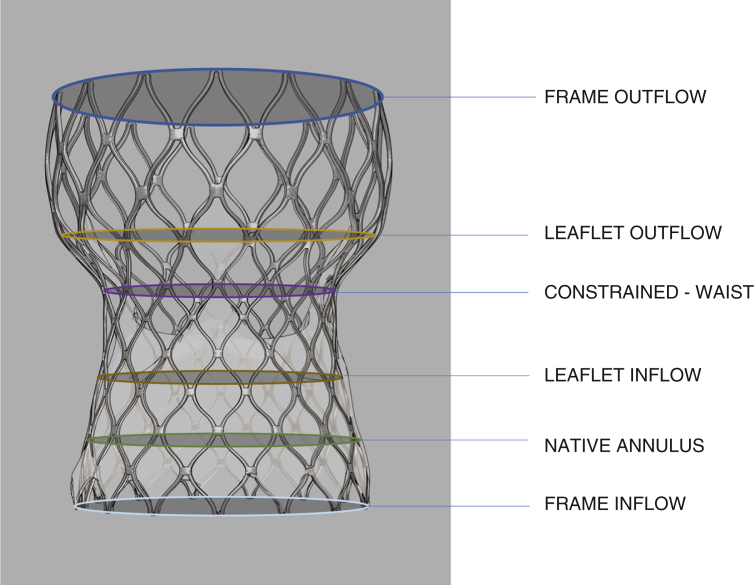
Eccentricity assessment at 6 prespecified prosthesis levels.

Eccentricity: The prosthesis eccentricity was calculated as previously reported^5^^,^^8^^,^^10^ at 6 prespecified prosthesis levels (Figure E1) as follows:

$$Eccentricity\;index=\sqrt{1-\frac{{\left(D\;\min\right)}^{2}}{{\left(Dmax\right)}^{2}}}$$

The eccentricity index is between 0 and 1. A larger eccentricity index (close to 1) represents a configuration similar to an oval shape, whereas a smaller index (close to 0) represents a circular orientation.^5^

**Figure E2:**
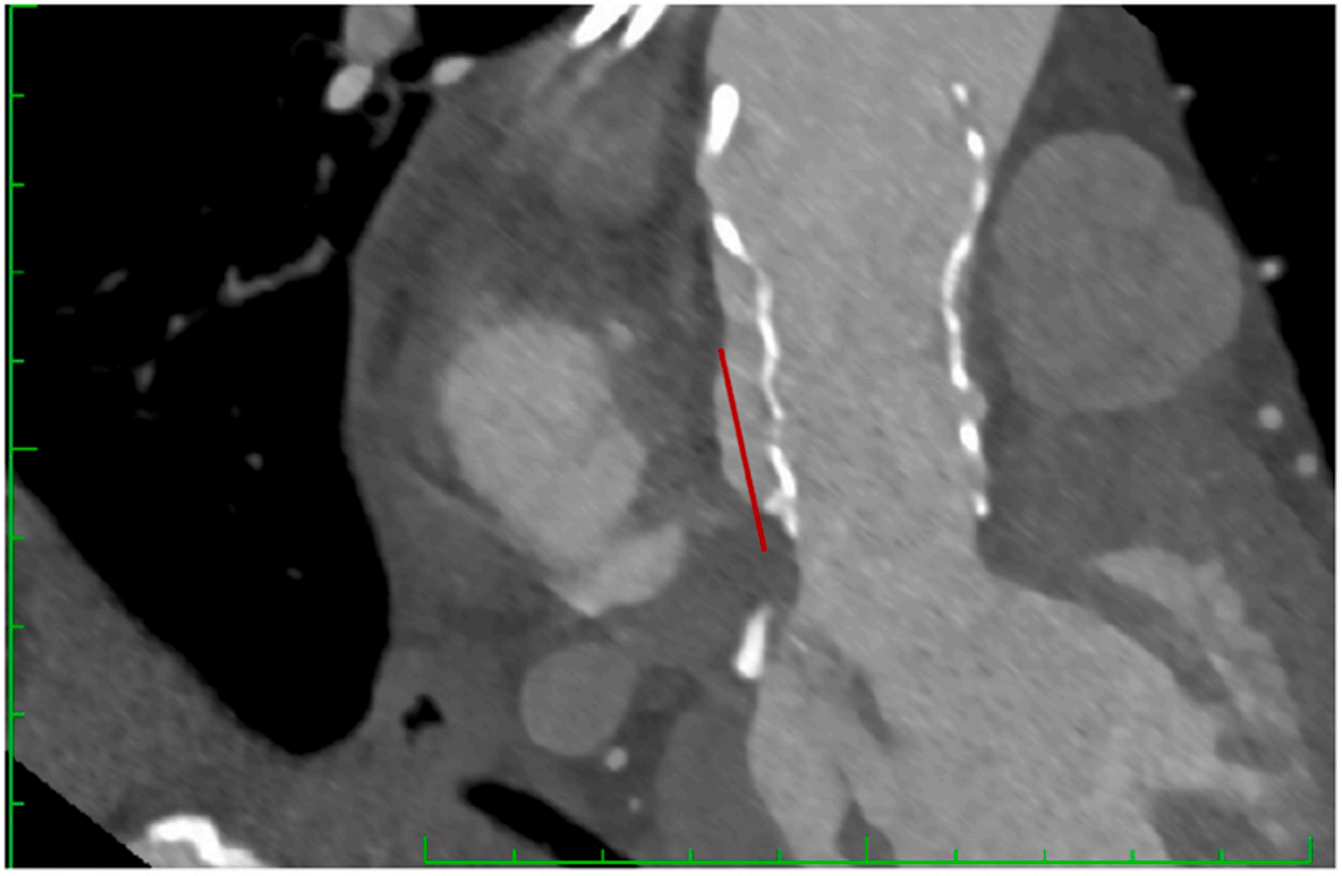
Schematic representation of implantation depth assessment.

Implantation depth: Because there is no specific recommendation in the Valve Academic Research Consortium-3 criteria,^18^ we measured the implantation depth by calculating, from the pre- and postimplant MDCT, the distance from the sinotubular junction and the virtual basal ring at the nadir of the left, right and noncoronary leaflet, and the distance from the sinotubular junction and the prosthesis frame inflow at the same nadir, as shown in Figure E2, *A* to *G*.^10^

**Figure E3:**
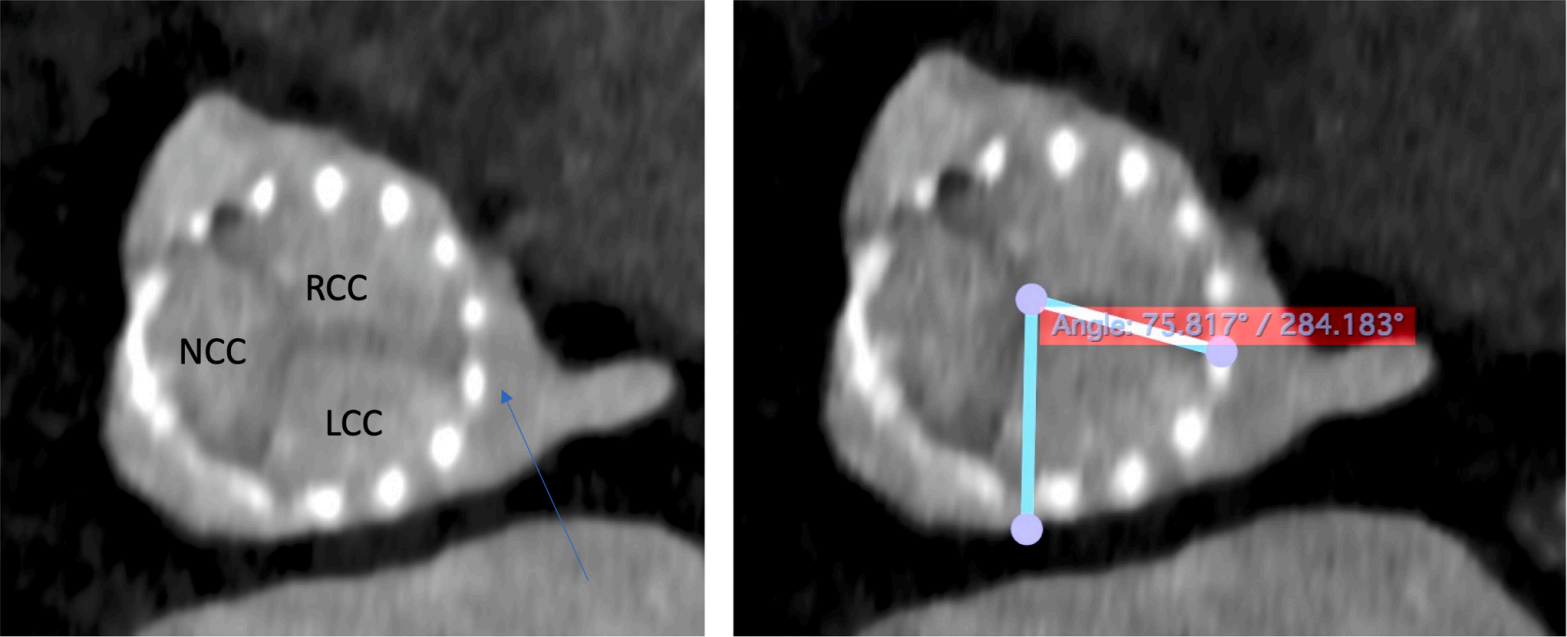
Example of severe commissural misalignment.

Commissural alignment was evaluated in both the native aortic valve (before implantation using MDCT) and the transcatheter heart valve (after implantation using MDCT), as described by others.^19^ By using the right coronary artery as a reference, an angle deviation of 30° or more was considered to be the cutoff for misalignment (anti-anatomic position) between the native and prosthetic valves (Figure E3).

Valve-to-coronary and noncoronary sinus distance was defined as the greatest distance measured from the prosthesis cage to the edge of the right and left coronary sinuses, respectively, and the noncoronary sinus.^20^

Morphology and classification of bicuspid valve: We used the Sievers and Schmidtke bicuspid morphological classification based on the number of raphes (type 0, I, and II) because it has been the most widely adopted.^21^ The decision to perform predilatation or postdilatation was based on the operator's judgment, considering factors such as annular size, calcification pattern, and degree of underexpansion seen during deployment.

### Anticoagulation and Antiplatelet Regimen

All patients received a prophylactic dose of unfractionated heparin throughout the hospital stay. During the procedure, unfractionated heparin was administered intravenously at a dose of 100 IU/kg. At discharge, patients were prescribed aspirin 100 mg daily unless dual antiplatelet therapy or oral anticoagulation was indicated for other conditions, such as coronary stenting or atrial fibrillation.

### Statistical Analysis

Missing data patterns were first visualized using a heatmap. Variables with more than 70% missing data were excluded from further analysis. For the remaining variables, numerical missing values were imputed using K-nearest neighbors within each training fold during cross-validation or within the training set of the held-out split. Any remaining gaps were addressed through median imputation. All preprocessing steps, including scaling, imputation, and class balancing, were carried out exclusively within training data to prevent any leakage of information into validation or test sets.

Normality of continuous variables was assessed using the Shapiro–Wilk test. Where distributions were clearly non-normal, variables were log-transformed or analyzed using nonparametric methods.

## Model Development

### 1. LASSO Logistic Regression

This penalized regression model was used to identify relevant predictors while reducing the risk of overfitting. L1 regularization helped shrink less informative coefficients to zero, effectively selecting a sparse set of predictors. The model was evaluated using area under the receiver operating characteristic curve (AUC-ROC, discrimination), Brier score (overall accuracy), and Hosmer–Lemeshow test to assess calibration.^13^

### 2. Random Forest Classifier

A Random Forest classifier was used to capture nonlinear relationships and interactions between variables. We tuned the number of trees, maximum tree depth, and minimum node size using grid search within cross-validation. Class imbalance was handled using the synthetic minority over-sampling technique (SMOTE) applied only to training data. Model performance was evaluated with out-of-bag error, 5-fold stratified cross-validation, and AUC-ROC on the held-out test set. Variable importance was assessed using the Gini index.^12^

### 3. XGBoost Model

The XGBoost model was trained using a randomized grid search with 5-fold cross-validation. We tuned hyperparameters including learning rate, maximum depth, child weight, and both L1 (alpha) and L2 (lambda) regularization terms. SMOTE was applied to training folds to address class imbalance. Model selection was based on AUC-ROC performance on the test set, with log-loss as a secondary criterion. SHapley Additive exPlanations (SHAP) values were used to evaluate the contribution of each variable to the model's predictions.^22^

## Model Performance Metrics

All models were evaluated using 5-fold stratified cross-validation and a separate 80/20 train-test split. Key performance metrics included (1) AUC-ROC for discrimination; (2) sensitivity, specificity, precision, accuracy, and F1-score for classification; and (3) confusion matrices for a visual understanding of class assignments. Calibration was assessed using the Brier score for all models, the Hosmer–Lemeshow test for LASSO, and calibration plots for Random Forest and XGBoost.^23^

### Validation Strategy and Overfitting Assessment

To reduce the risk of overfitting and ensure generalizability, all preprocessing steps (imputation, scaling, SMOTE) were limited to training data. Stratified 5-fold cross-validation was used to maintain class proportions in each fold. We compared performance across folds and against the held-out test set. Simpler models and those with regularization, such as LASSO, were favored when performance was comparable.

### Class Imbalance Handling

To address potential class imbalance (ie, different prevalence of SLT), we used SMOTE to synthetically balance the training data in the Random Forest and XGBoost models. Stratified cross-validation was used throughout to ensure consistent SLT/non-SLT ratios in all folds. Performance metrics like F1-score, precision, and sensitivity were prioritized over accuracy to better reflect the model's ability to capture the minority class.

### Feature Importance Analysis

To interpret model predictions, we assessed: variable importance plots from LASSO (magnitude of non-zero coefficients), Random Forest (Gini index), and XGBoost (SHAP values).

### Statistical Environment

All statistical analyses and ML models were performed using R (R Studio Version 2024.12.0) with the following packages^24^: tidyverse, mice, stats, MASS, ResourceSelection, randomForest, xgboost, caret, pROC, MLmetrics, and ggplot2.

## Results

### Patients’ Baseline Characteristics and Periprocedural Details

**Figure 1:**
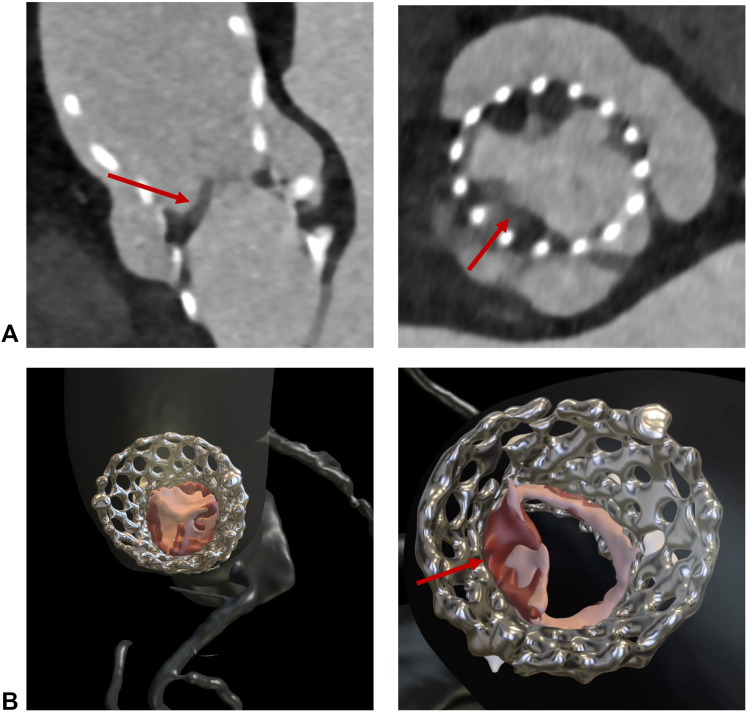
A case of subclinical thrombosis determining moderate RELM visualized with (A) MDCT and (B) multiparametric digital approach. *MDCT,* Multidetector computed tomography.

Subclinical leaflet thrombosis, from mild to severe, was detected at the 6-month MDCT scan (mean 182 days, SD) ± 12 days) in 22 patients (Figure 1 and Video 1). Patients’ characteristics and periprocedural details for the overall population and stratified by SLT and No-SLT are shown in Table 1. Burden of SLT/HALT and RELM is shown in Table 2. Echocardiographic and MDCT details are shown in Tables E2 and E3. There was no difference between use of oral anticoagulants or dual antiplatelet therapy between the SLT and No-SLT groups before implantation. At the time of the MDCT scan, there were also no significant differences in antithrombotic regimens between the SLT and No-SLT groups. Most patients were receiving single antiplatelet therapy (80.6%), and the use of direct oral anticoagulants (11.5%) and oral anticoagulation (8.7%) was similar across SLT and No-SLT (Table E4). A total of 21 patients (17.8%) had bicuspid aortic valve anatomy.

**Table 1:** Characteristics of patients at baseline and periprocedural details

	Overall cohort N = 118	SLT		*P* value
		No N = 96	Yes N = 22	
Age, y	78.4 (5.7)	78.1 (5.7)	79.6 (5.8)	.263
BSA,∗ m^2^	1.8 (0.2)	1.8 (0.2)	1.8 (0.2)	.737
BMI†	28.1 (5.0)	27.8 (4.5)	29.3 (6.8)	.200
BMI ≥ 30	31 (26.5)	24 (25.3)	7 (31.8)	.719
Male sex, no. (%)	62 (6.2)	53 (55.2)	9 (40.9)	.330
EuroSCORE II, %	1.8 (1.8)	1.8 (1.7)	1.8 (1.7)	.222
Low flow – low gradient, n (%)	12 (11.5)	9 (10.5)	3 (16.7)	.731
NYHA functional class III-IV, no. (%)	14 (13.9)	14 (16.9)	0	.133
NIDDM/IDDM, no. (%)	37 (31.9)	30 (31.6)	7 (33.3)	.999
Hypertension, no. (%)	114 (98.3)	94 (98.9)	20 (95.2)	.798
COPD, no. (%)	19 (17.8)	15 (16.9)	4 (22.2)	.837
Previous stroke/TIA, no. (%)	6 (5.6)	6 (6.7)	0	.567
Previous PCI, no. (%)	24 (22.4)	21 (23.6)	3 (16.7)	.739
Previous cardiac surgery, no. (%)	10 (9.4)	8 (9.1)	2 (11.1)	.988
Previous MI, no. (%)	20 (18.7)	17 (19.1)	3 (16.7)	.997
Coronary artery disease, no. (%)	30 (28.3)	25 (28.4)	5 (27.8)	.776
Serum creatinine, mg/dL	0.8 (0.2)	0.8 (0.4)	0.8 (0.7)	.659
Creatinine clearance by Cockcroft-Gault formula (mL/min)	67 (48.1)	67 (37.2)	68.2 (49.5)	.713
Pre-existing pacemaker or defibrillator, no. (%)	11 (11.0)	7 (8)	4 (22.2)	.166
History of right bundle-branch block, no. (%)	3 (3.0)	2 (2.4)	1 (5.6)	.999
Bicuspid valve	16 (13.3)	6 (37.5)	10 (62.5)	<.001
Procedural characteristics				.422
Valve type				
Evolut R	105 (89.0)	86 (89.6)	19 (86.4)	
Portico	8 (6.8)	7 (7.3)	1 (4.5)	
Navitor	5 (4.2)	3 (3.1)	2 (9.1)	
Valve size				.582
23 mm	10 (8.5)	9 (9.5)	1 (4.5)	
25 mm	3 (2.5)	2 (2.1)	1 (4.5)	
26 mm	45 (38.5)	37 (38.9)	8 (36.4)	
27 mm	5 (4.3)	3 (3.2)	2 (9.1)	
29 mm	39 (33.3)	31 (32.6)	8 (36.4)	
34 mm	16 (13.7)	14 (14.7)	2 (9.1)	
Pre-TAVR balloon valvuloplasty	37 (31.4)	32 (33.3)	5 (22.7)	.476
Post-TAVR balloon valvuloplasty	51 (43.2)	41 (42.7)	10 (45.5)	.989

Values are reported as the mean and SD or number and percentage (%). *SLT,* Subclinical leaflet thrombosis; *BSA,* body surface area; *BMI,* body mass index; *EuroSCORE,* European System for Cardiac Operative Risk Evaluation; *NYHA,* New York Heart Association; *NIDDM,* noninsulin-dependent diabetes mellitus; *IDDM,* insulin-dependent diabetes mellitus; *COPD,* chronic obstructive pulmonary disease; *TIA,* transient ischemic attack; *PCI,* percutaneous coronary intervention; *MI,* myocardial infarction; *TAVR,* transcatheter aortic valve replacement.

∗ Body surface area was calculated with the Du Bois formula: 0.007 × Weight^0.425^ × Height^0.725^.

† The body mass index was calculated as the weight in kilograms divided by the square of the height in meters.

**Table 2:** Burden of subclinical leaflet thrombosis and restricted leaflet movement

N = 118	
Patient with leaflet thrombosis, n (%)∗	22 (18.6)
Involvement of a single leaflet	10 (8.4)
Involvement of 2 leaflets	5 (4.2)
Involvement of 3 leaflets	7 (5.9)
Hypoattenuated lesions leaflet n (%)	
HALT ≤ 25%	10 (8.4)
HALT >25%-50%	21 (17.7)
HALT >50%-75%	2 (1.7)
HALT >75%	8 (6.7)
RELM (%)	
RELM ≤ 25%	19 (16.1)
RELM >25%-50%	20 (16.9)
RELM >50%-75%	2 (1.69)
RELM >75%	0

Values are reported as number and percentage (%). *HALT,* Hypoattenuated leaflet thickening; *RELM,* restricted leaflet movement.

∗ One patient may have more than 1 of SLT/HALT simultaneously.

### Predictors of Subclinical Leaflet Thrombosis

**Figure 2:**
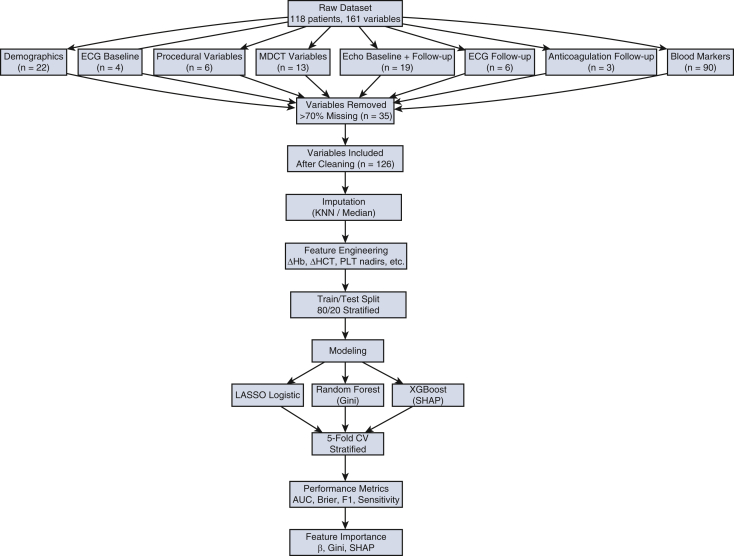
ML pipeline.

**Figure E4:**
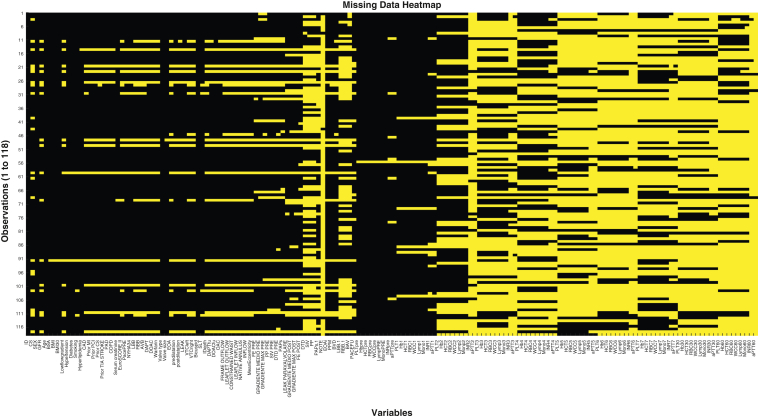
Heatmap, missing variables.

Variables initially included (N = 161) and percentage of missing data are presented in the heatmap in Figure E4 and Table E1. A total of 35 variables had missing data more than 70%, the vast majority of which were postoperative variables, and were removed. The final multimodeling analysis was then performed on 126 variables (Figure 2).

### Logistic Regression Model

**Figure 3:**
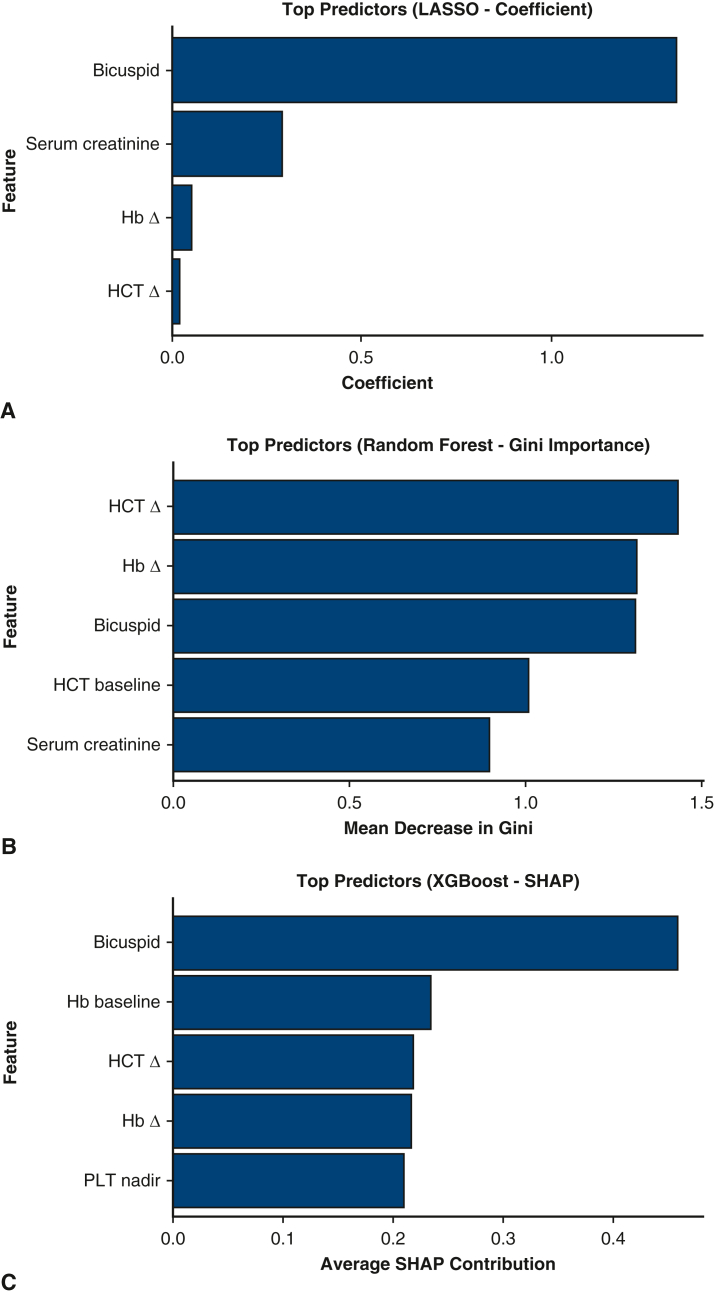
Top predictors of SLT. A, LASSO logistic regression predictors of SLT based on non-zero coefficients: bicuspid valve morphology (β = 1.33), serum creatinine (β = 0.29), hemoglobin decrease (ΔHb) (β = 0.05), and hematocrit decrease (ΔHCT) (β = 0.02). B, Random Forest top 5 predictors using Gini importance: ΔHCT (Gini: 1.43), ΔHb (1.32), bicuspid valve morphology (1.31), baseline hematocrit (1.01), and serum creatinine (0.90). C, XGBoost model top predictors (SHAP): bicuspid valve morphology (SHAP: 0.42), baseline hemoglobin (SHAP: 0.16), hematocrit decrease (SHAP: 0.11), hemoglobin decrease (SHAP: 0.10), and platelet nadir (SHAP: 0.09). *Hb,* Hemoglobin; *HCT,* hematocrit; *LASSO,* least absolute shrinkage and selection operator; *PLT,* platelet; *SHAP,* SHapley Additive exPlanations; *SLT,* subclinical leaflet thrombosis; *XGBoost,* Extreme Gradient Boosting.

By using a LASSO logistic regression model, we identified 4 key variables associated with the occurrence of subclinical leaflet thrombosis (SLT). The strongest predictor was bicuspid valve morphology (β = 1.33), followed by elevated serum creatinine (β = 0.29), perioperative hemoglobin decrease (ΔHb) (β = 0.05), and hematocrit decrease (ΔHCT) (β = 0.02) (Figure 3, *A*).

**Figure 4:**
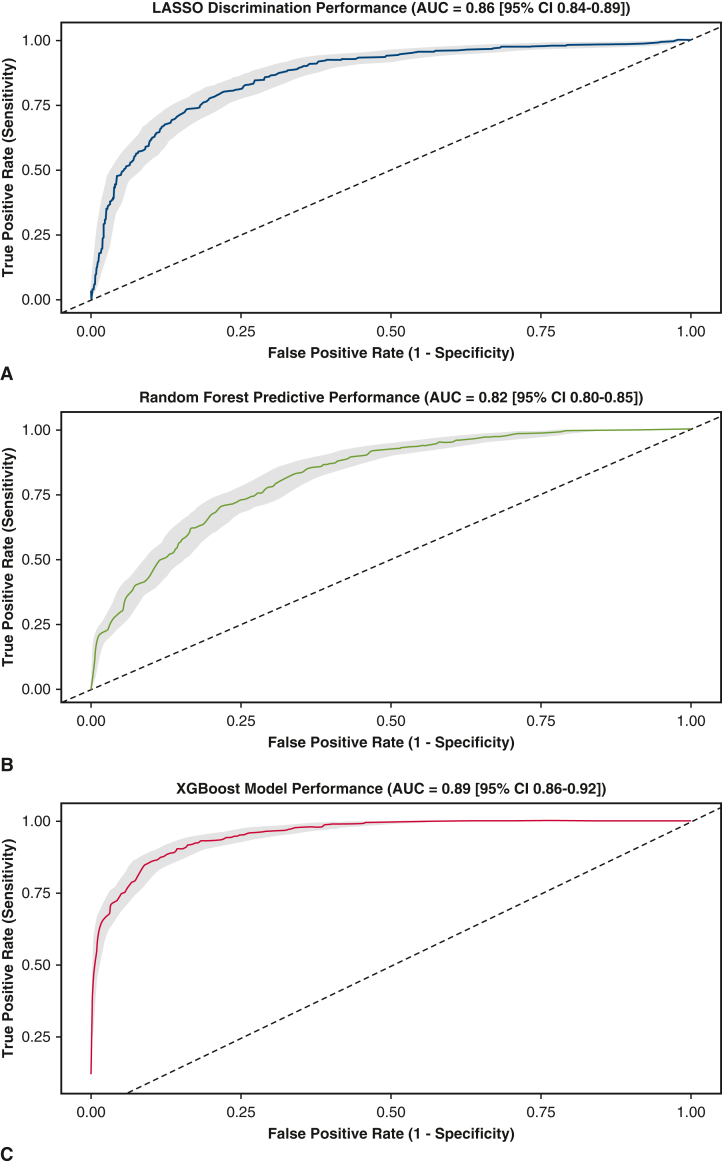
Discriminative performance of ML models for predicting SLT. A, ROC curve of the LASSO logistic regression model with AUC of 0.86 (95% CI, 0.84-0.99). B, ROC curve of the Random Forest model trained on a SMOTE-balanced dataset with AUC of 0.82 (95% CI, 0.80-0.85). C, ROC curve of the XGBoost model with an AUC of 0.89 (95% CI, 0.86-0.92). *AUC,* Area under the curve; *LASSO,* least absolute shrinkage and selection operator; *ROC,* receiver operating characteristic; *SLT,* subclinical leaflet thrombosis; *SMOTE,* Synthetic Minority Oversampling Technique; *XGBoost,* Extreme Gradient Boosting.

The model demonstrated good discriminative performance, with an AUC-ROC of 0.86 (95% CI, 0.84-0.99; Figure 4, *A*). Calibration appeared acceptable, as reflected by a low Brier score of 0.040, although the Hosmer–Lemeshow test showed a borderline result (chi-square = 15.27, df = 8, *P* = .054), suggesting potential room for improvement. At the optimal probability threshold, the model yielded a sensitivity of 0.75, specificity of 0.625, precision of 0.50, accuracy of 0.67, and F1-score of 0.60.

### Random Forest Model

**Figure E5:**
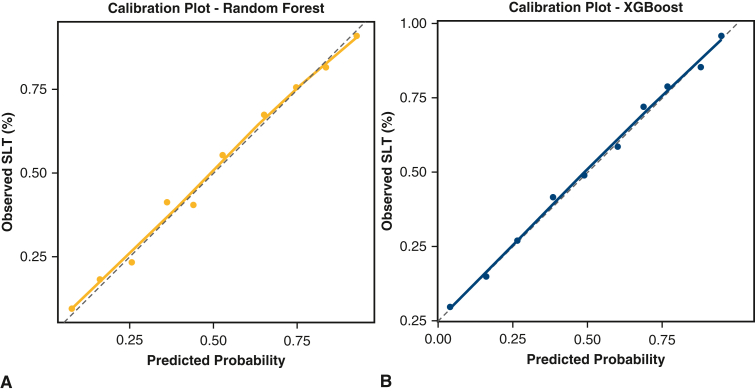
A and B, Calibration plot.

Based on Gini importance, the 5 most influential predictors were the decrease in hematocrit from baseline to postoperative nadir (ΔHCT, Gini: 1.43), hemoglobin decrease (ΔHb, Gini: 1.32), bicuspid valve morphology (Gini: 1.31), baseline hematocrit (Gini: 1.01), and serum creatinine (Gini: 0.90) (Figure 3, *B*). The model yielded an out-of-bag error rate of 15%, suggesting good internal consistency. When applied to an independent test set, its performance remained stable, with an AUC of 0.84 (95% CI, 0.80-0.85; Figure 4, *B*). The classification error for SLT cases was 11.8%, reflecting the model's ability to identify minority-class events. At the optimal probability threshold, sensitivity reached 0.81, with a specificity of 0.70, a precision of 0.73, an overall accuracy of 0.76, and an F1-score of 0.77. The Brier score was 0.163, and the corresponding calibration plot is shown in Figure E5, *A* and *B*.

### XGBoost Model

**Figure 5:**
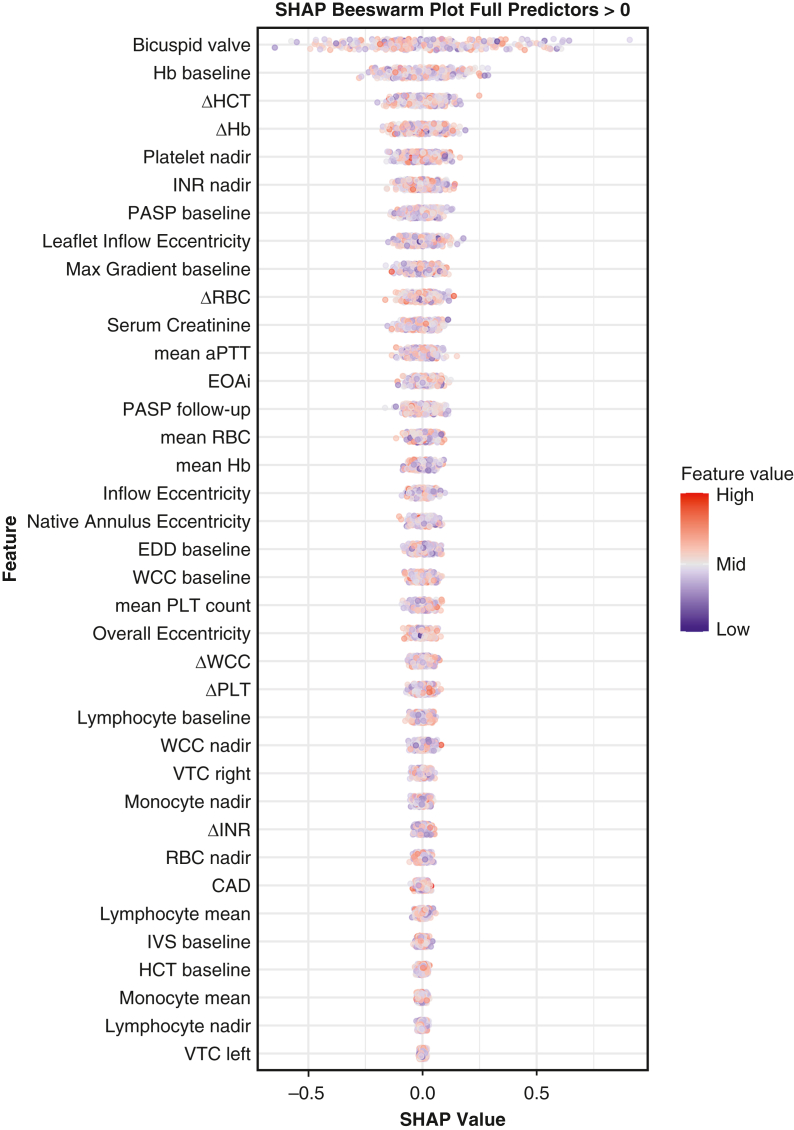
Beeswarm plot based on the analysis of 120 features showing all the factors (n = 37) associated with SLT with mean SHAP value different from 0. *aPTT,* Activated partial thromboplastin time; *CAD,* coronary artery disease; *EDD,* end-diastolic diameter; *EOAi,* indexed effective orifice area; *Hb,* hemoglobin; *HCT,* hematocrit; *INR,* international normalized ratio; *IVS,* interventricular septum; *PASP,* pulmonary artery systolic pressure; *PLT,* platelet; *RBC,* red blood cell; *SHAP,* SHapley Additive exPlanations; *SLT,* subclinical leaflet thrombosis; *VTC,* valve-to-coronary (left/right) (see Table E2).

In the SHAP analysis, the top 5 predictors of SLT at 6-month follow-up were bicuspid aortic valve morphology (mean SHAP: 0.42), baseline hemoglobin (mean SHAP: 0.16), decrease in hematocrit (mean SHAP: 0.11), decrease in hemoglobin (mean SHAP: 0.10), and platelet nadir (mean SHAP: 0.09) (Figure 3, *C*). The XGBoost model demonstrated excellent discriminative performance, achieving an AUC of 0.89 (95% CI, 0.86-0.92) on the test set (Figure 4, *C*). Test set metrics included sensitivity of 0.83, specificity of 0.88, precision of 0.77, accuracy of 0.84, and F1-score of 0.71. The Brier score was 0.131, and calibration was visually assessed (Figure E5, *A* and *B*). All predictors with non-zero SHAP values (n = 37) are shown in the beeswarm plot (Figure 5) and Table E5.

## Discussion

**Figure 6:**
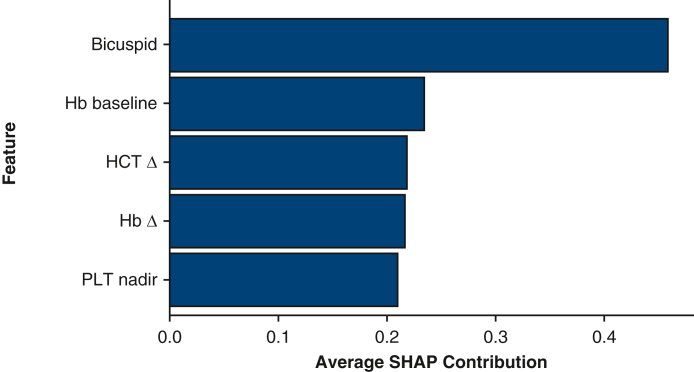
Central Picture. Machine learning derived predictor of subclinical leaflet thrombosis.

In this study, we identified the top predictors of SLT across 3 ML approaches: LASSO, Random Forest, and XGBoost. Variable selection was based on non-zero coefficients (LASSO), Gini importance (Random Forest), and SHAP values (XGBoost). Notably, all 3 models consistently identified native aortic bicuspid valve stenosis as one of the most influential predictor of SLT at the 6-month follow-up. In particular, SHAP analysis in the XGBoost model demonstrated that bicuspid valve morphology had the highest mean SHAP value by a considerable margin, accounting for the largest individual contribution to model output (Figure 6). TAVI for bicuspid aortic stenosis remains challenging and has been associated with suboptimal outcomes at follow-up.^25^ Anatomic deviations from normal postimplant prosthesis geometry may adversely affect valve performance, disrupt laminar blood flow, elevate shear stress, and increase the propensity for thrombus formation.^26^^,^^27^

The link between bicuspid valve anatomy and a higher rate of SLT is particularly important, given that more relatively young patients are now being treated with TAVI.^25^ This could play a role in reduced long-term valve durability.

In addition, hematological markers, including preoperative hemoglobin levels, hemoglobin and hematocrit decreases, and platelet nadir, were recurrently selected across models, suggesting a potential interplay between postoperative hematologic shifts and thrombotic leaflet dysfunction.

This is hypothesis generating, for example, postimplant thrombocytopenia is a common yet poorly understood phenomenon,^28^ and our findings may underscore a previously unrecognized link between SLT and dynamic hematological changes after valve implantation.

As such, monitoring hematological changes in the early postoperative period, while flagging patients with bicuspid valve morphology, may help identify individuals at increased risk of SLT and could inform future strategies regarding the use of anticoagulant therapy.

To the best of our knowledge, this is the first study to explore predictors of SLT using a combination of different ML methods. These approaches are becoming increasingly used in clinical research because they can manage nonlinear relationships and reduce reliance on strict statistical assumptions.^9^ This is particularly helpful when studying complex conditions like SLT, which involve multiple interacting factors and are often investigated in modestly sized cohorts.^9^

Beyond the primary 5 variables, additional predictors with non-negligible SHAP values included international normalized ratio nadir, baseline pulmonary artery systolic pressure, leaflet inflow eccentricity, and native annulus eccentricity. Alongside the main hematological markers, additional variables such as ΔRBC, mean aPTT, and baseline white cell count were also retained in the model, although with lesser weight. These findings point to a possible link between postoperative hematological shifts and the risk of SLT. However, this analysis did not include derived inflammatory ratios such as the lymphocyte-to-monocyte ratio or neutrophil-to-lymphocyte ratio, nor were biomarkers such as (B-type natriuretic peptide) or interleukins assessed. These markers could be relevant and warrant investigation in larger cohorts moving forward.

Among the MDCT-derived measurements, including eccentricity, implantation depth, commissural alignment, and valve-to-coronary distance, none ranked among the top 5 predictors of SLT. However, bicuspid valve morphology, which emerged as the strongest predictor, has been linked to prosthesis deformation and nonuniform expansion, potentially influencing thrombotic risk.^15^

### Limitations

The main limitation of this study lies in the relatively limited sample size; however, few studies to date have achieved such a consistent number of longitudinal MDCT assessments at 6-month follow-up, particularly in the context of self-expanding prostheses.

Also, balloon-expandable prostheses were not included in the analysis. This cohort predominantly consisted of relatively low-risk patients, the vast majority of whom were in preoperative sinus rhythm and had no indication for oral anticoagulation due to nonvalvular conditions. As a result, the effect of oral anticoagulation on SLT could not be assessed.

Only selected baseline and postimplant MDCT parameters were included in the analysis; a more comprehensive model incorporating additional detailed MDCT features might have yielded more specific or nuanced results. Although paravalvular leak was included in our multivariable analysis, it was not independently associated with the occurrence of SLT. Nonetheless, we cannot fully exclude the possibility that paravalvular leak may contribute indirectly through mechanisms such as postprocedural hemolysis or anemia, which could in turn influence hematologic markers relevant to SLT pathophysiology.

Patient recruitment was slower in the early study period and limited by factors such as renal function, atrial fibrillation, or refusal to undergo MDCT. These constraints may have introduced selection bias and affected the generalizability of our findings. The effect of Sievers classification on SLT risk could not be assessed in a granular manner due to limited availability and low representation of specific bicuspid subtypes.

Inter-operator variability for MDCT measurements was not formally assessed via intraclass correlation coefficient, which may limit the reproducibility of imaging-based findings.

Surgeon and institutional volume were not included as predictors, because all procedures were performed in a single high-volume center, potentially limiting external generalizability.

Long-term clinical follow-up beyond the 6-month MDCT scan was not available, limiting assessment of late outcomes.

Another limitation of this study is the absence of time-aware ML models, which could have accounted for the dynamic trends in blood markers collected during the first week postimplantation. Although our approach captures key baseline predictors and integrates early postprocedural biomarkers, future studies incorporating temporal modeling techniques (eg, recurrent neural networks or time-dependent gradient boosting) may provide deeper insights into the evolving risk of SLT. Although XGBoost yielded excellent predictive performance in our study, it remains a complex, nonlinear ensemble method that is often referred to as a “black box” model.^29^ Given the limited number of SLT events (n = 22) and the high dimensionality of the feature space, we acknowledge that the reported metrics may be optimistic and should be interpreted with caution. These findings warrant future external validation in larger cohorts.

## Conclusions

In patients treated with self-expanding TAVI valves, bicuspid valve anatomy was consistently associated with a higher risk of SLT at 6 months. Hematological markers, such as baseline hemoglobin, its postoperative decline, and platelet nadir, also showed a reproducible association with SLT across all models. These are values that are routinely available in clinical practice and, when considered together with anatomic characteristics, may help identify patients at increased risk.

This becomes particularly relevant because TAVI is now increasingly performed in younger individuals, many of whom present with bicuspid valve disease. In these patients, prosthesis performance and long-term durability are essential. Identifying early signs that may compromise valve function, such as SLT, could help tailor follow-up imaging and guide the use of antithrombotic therapy. These findings support the need for further prospective studies to confirm the results and assess whether they can inform clinical decision-making in everyday practice.

## Conflict of Interest Statement

The authors reported no conflicts of interest.

The *Journal* policy requires editors and reviewers to disclose conflicts of interest and to decline handling or reviewing manuscripts for which they may have a conflict of interest. The editors and reviewers of this article have no conflicts of interest.

## Figures and Tables

**Table E1:** Variables considered and missing data summary

Variable N = 161	Missing values	Percentage
Demographic n = 22		
ID	0.0	0.00%
Calcium score	22.0	18.64%
Sex/gender	0.0	0.00%
GFR	0.0	0.00%
Age	0.0	0.00%
BSA	1.0	0.85%
BMI	1.0	0.85%
BMI >30	1.0	0.85%
Low flow gradient	14.0	11.86%
Hypertension	2.0	1.69%
Diabetes	2.0	1.69%
Smoking	4.0	3.39%
Hyperlipidemia	3.0	2.54%
Coronary artery disease	12.0	10.17%
Prior MI	11.0	9.32%
Prior PCI	11.0	9.32%
Prior CS	12.0	10.17%
Prior TIA/stroke	11.0	9.32%
PAD	11.0	9.32%
COPD	11.0	9.32%
Serum creatinine	7.0	5.93%
EuroSCORE II	17.0	14.41%
NYHA III-IV	12.0	10.17%
ECG details at baseline n = 4		
Permanent pacemaker	12.0	10.17%
LBB	17.0	14.41%
RBB	17.0	14.41%
AVB	17.0	14.41%
Procedural n = 6		
Valve type	0.0	0.00%
Valve size	1.0	0.85%
EOA	18.0	15.25%
Predilatation	11.0	9.32%
Postdilatation	12.0	10.17%
Leak (angio evaluation)	11.0	9.32%
MDCT (baseline and follow-up 6 mo) n = 13		
VTC distance left	0.0	0.00%
VTC distance right	0.0	0.00%
Bicuspid	0.0	0.00%
SLT	0.0	0.00%
ID depth	0.0	0.00%
Eccentricity		
Frame outflow	0.0	0.00%
Leaflet outflow	0.0	0.00%
Constrained/waist	0.0	0.00%
Leaflet inflow	0.0	0.00%
Native annulus	0.0	0.00%
Frame inflow	0.0	0.00%
Mean eccentricity	0.0	0.00%
Commissural misalignment	0.0	0.00%
Echocardiographic n = 19		
LVEF baseline	18.0	15.25%
Mean gradient baseline	20.0	16.95%
Max gradient baseline	23.0	19.49%
Posterior wall baseline	21.0	17.80%
IVS baseline	21.0	17.80%
EDD baseline	24.0	20.34%
PASP baseline	22.0	18.64%
Paravalvular leak	22.0	18.64%
Mean gradient follow-up	24.0	20.34%
Max gradient follow-up	25.0	21.19%
LVEF follow-up	26.0	22.03%
EDD follow-up	71.0	60.17%
IVS follow-up	71.0	60.17%
Posterior wall follow-up	71.0	60.17%
PASP follow-up	54.0	45.76%
EOAi	15.0	12.71%
Patient prosthesis mismatch	16.0	13.56%
Bioprosthesis valve dysfunction	18.0	15.25%
ECG details at follow-up n = 4		
LBB	16.0	13.56%
RBB	15.0	12.71%
Atrio-ventricular block	15.0	12.71%
Pacemaker at follow-up	0.0	0.00%
Anticoagulation status at follow-up n = 3		
DAPT	0.0	0.00%
DOAC	0.0	0.00%
OAC	0.0	0.00%
Blood markers n = 90		
PLTpre	1.0	0.85%
Hbpre	1.0	0.85%
HCTpre	1.0	0.85%
RBCpre	1.0	0.85%
WCCpre	1.0	0.85%
LympPRE	1.0	0.85%
MonoPRE	1.0	0.85%
INRpre	8.0	6.78%
aPTTpre	8.0	6.78%
PLT1	6.0	5.08%
Hb1	6.0	5.08%
HCT1	6.0	5.08%
RBC1	6.0	5.08%
WCC1	6.0	5.08%
Lymp1	6.0	5.08%
Mono1	6.0	5.08%
INR1	15.0	12.71%
aPTT1	14.0	11.86%
PLT2	41.0	34.75%
Hb2	41.0	34.75%
HCT2	41.0	34.75%
RBC2	41.0	34.75%
WCC2	41.0	34.75%
Lymp2	41.0	34.75%
Mono2	41.0	34.75%
INR2	103.0	87.29%
aPTT2	103.0	87.29%
PLT3	64.0	54.24%
Hb3	64.0	54.24%
HCT3	64.0	54.24%
RBC3	64.0	54.24%
WCC3	64.0	54.24%
Lymp3	64.0	54.24%
Mono3	64.0	54.24%
INR3	87.0	73.73%
aPTT3	85.0	72.03%
PLT4	60.0	50.85%
Hb4	60.0	50.85%
HCT4	60.0	50.85%
RBC4	60.0	50.85%
WCC4	60.0	50.85%
Lymp4	60.0	50.85%
Mono4	60.0	50.85%
INR4	74.0	62.71%
aPTT4	74.0	62.71%
PLT5	80.0	67.80%
Hb5	80.0	67.80%
HCT5	80.0	67.80%
RBC5	80.0	67.80%
WCC5	80.0	67.80%
Lymp5	80.0	67.80%
Mono5	80.0	67.80%
INR5	93.0	78.81%
aPTT5	93.0	78.81%
PLT6	89.0	75.42%
Hb6	89.0	75.42%
HCT6	89.0	75.42%
RBC6	89.0	75.42%
WCC6	89.0	75.42%
Lymp6	89.0	75.42%
Mono6	89.0	75.42%
INR6	102.0	86.44%
aPTT6	103.0	87.29%
PLT7	69.0	58.47%
Hb7	69.0	58.47%
HCT7	69.0	58.47%
RBC7	69.0	58.47%
WCC7	69.0	58.47%
Lymp7	69.0	58.47%
Mono7	69.0	58.47%
INR7	85.0	72.03%
aPTT7	85.0	72.03%
PLT30	84.0	71.19%
Hb30	84.0	71.19%
HCT30	84.0	71.19%
RBC30	84.0	71.19%
WCC30	84.0	71.19%
Lymp30	84.0	71.19%
Mono30	84.0	71.19%
INR30	92.0	77.97%
aPTT30	92.0	77.97%
PLT60	87.0	73.73%
Hb60	87.0	73.73%
HCT60	87.0	73.73%
RBC60	88.0	74.58%
WCC60	88.0	74.58%
Lymp60	88.0	74.58%
Mono60	89.0	75.42%
INR60	113.0	95.76%
aPTT60	115.0	97.46%

BMI was calculated as the weight in kilograms divided by the square of the height in meters.

Columns and variables with more than 70% missing value were removed.

Columns with >70% missing data (n = 35).

[1] “INR2” “aPTT2” “INR3” “aPTT3” “INR5” “aPTT5” “PLT6” “Hb6”

[9] “HCT6” “RBC6” “WCC6” “Lymp6” “Mono6” “INR6” “aPTT6” “INR7”

[17] “aPTT7” “PLT30” “Hb30” “HCT30” “RBC30” “WCC30” “Lymp30” “Mono30”

[25] “INR30” “aPTT30” “PLT60” “Hb60” “HCT60” “RBC60” “WCC60” “Lymp60”

[33] “Mono60” “INR60” “aPTT60”

*ID,* Implantation depth; *GFR,* glomerular filtration rate; *BSA,* body surface area; *BMI,* body mass index; *MI,* myocardial infarction; *PCI,* percutaneous coronary intervention; *CS,* cardiac surgery; *TIA,* transient ischemic attack; *PAD,* peripheral artery disease; *COPD,* chronic obstructive pulmonary disease; *NYHA,* New York Heart Association; *LBB,* left bundle branch block; *RBB,* right bundle branch block; *AVB,* atrioventricular block; *VTC,* valve-to-coronary; *SLT,* subclinical leaflet thrombosis; *LVEF,* left ventricular ejection fraction; *IVS,* interventricular septum; *EDD,* end-diastolic diameter; *PASP,* pulmonary artery systolic pressure; *EOAi,* effective orifice area indexed; *DAPT,* dual antiplatelet therapy; *DOAC,* direct oral anticoagulant; *OAC,* oral anticoagulation; *PLT,* platelet; *RBC,* red blood cells; *WCC,* white cell count; *Lymph,* lymphocyte; *Mono,* monocyte. BSA was calculated with the Du Bois formula: 0.007 × Weight 0.425 × Height 0.725.

**Table E2:** Echocardiographic characteristics according to subclinical leaflet thrombosis

	Overall cohort N = 118	SLT		*P* value
		No N = 96	Yes N = 22	
Baseline				
LVEF %, mean ± SD	54.04 (10.31)	53.02 (11.53)	55.30 (8.53)	.28
LVEDD mm, mean ± SD	45.84 (7.28)	46.55 (7.36)	44.94 (7.16)	.29
IVS mm, mean ± SD	13.43 (2.47)	13.32 (2.86)	13.56 (1.90)	.64
PWT mm, mean ± SD	12.41 (2.28)	12.53 (2.43)	12.26 (2.09)	.56
AVMG mm Hg, mean ± SD	47.99 (13.18)	46.10 (10.69)	50.33 (15.55)	.12
AVPG mm Hg, mean ± SD	80.52 (74.76)	82.69 (98.66)	77.83 (23.13)	.75
PASP mm Hg, mean ± SD	37.27 (10.89)	37.53 (11.50)	36.95 (10.22)	.80
At discharge				
LVEF %, mean ± SD	55.21 (6.71)	55.25 (7.10)	55.23 (6.60)	.68
LVEDD mm, mean ± SD	45.74 (7.21)	46.55 (7.35)	44.90 (7.11)	.29
IVS mm, mean ± SD	13.42 (2.47)	13.33 (2.86)	13.01 (1.91)	.63
PWT mm, mean ± SD	12.40 (2.28)	12.55 (2.42)	12.20 (2.01)	.56
AVMG mm Hg, mean ± SD	6.96 (3.05)	7.1 (3.02)	6.6 (3.08)	.35
AVPG mm Hg, mean ± SD	12.36 (5.11)	12.80 (5.59)	12.80 (6.73)	.55
PASP mm Hg, mean ± SD	37.01 (10.11)	37.03 (11.40)	36.84 (10.01)	.69
EOA cm^2^	1.85 (0.29)	1.85 (0.31)	1.87 (0.41)	.91
EOAi cm^2^	1.03 (0.15)	1.03 (0.16)	1.04 (0.16)	.93
At follow-up				
LVEF %, mean ± SD	55.61 (6.81)	55.35 (7.03)	55.92 (6.60)	.69
LVEDD mm, mean ± SD	46.89 (20.50)	48.40 (27.10)	45.89 (4.09)	.58
IVS mm, mean ± SD	11.06 (1.33)	11.30 (1.34)	10.74 (1.28)	.16
PWT mm, mean ± SD	11.45 (5.90)	10.96 (1.90)	12.11 (8.81)	.53
AVMG mm Hg, mean ± SD	7.12 (3.59)	7.23 (3.14)	6.97 (4.16)	.73
AVPG mm Hg, mean ± SD	13.47 (6.10)	13.81 (5.62)	13.00 (6.75)	.53
PASP mm Hg, mean ± SD	33.92 (6.98)	33.86 (7.22)	34.00 (6.77)	.94
Prosthetic aortic valve regurgitation				.42
None/trace	71 (60.1)	59 (61.4)	12 (54.5)	
Mild	38 (32.2)	29 (30.2)	9 (40.9)	
Moderate	9 (7.6)	8 (8.3)	1 (4.5)	
Severe	0	0	0	
EOA cm^2^	1.87 (0.38)	1.86 (0.33)	1.87 (0.40)	.92
EOAi cm^2^	1.03 (0.13)	1.03 (0.14)	1.04 (0.12)	.90
PPM∗ n (%)				.98
Moderate	8 (6.7)	7 (7.2)	1 (4.5)	
Severe	0			
Increased 10 mm Hg gradient from discharge, n (%)	1 (0.8)	0	1 (4.5)	
Endocarditis, n (%)	0			
Clinical thrombosis, n (%)	0			
HSVD n, (%)	1 (0.8)	0	1 (4.5)	
BVD n, (%)	17 (14.4)	15 (15.5)	2 (9)	.76

Values are reported as the mean and SD or number and percentage (%). *SLT*, Subclinical leaflet thrombosis; *LVEF*, left ventricle ejection fraction; *LVEDD*, left ventricular end-diastolic diameter; *IVS*, interventricular septum; *PWT*, posterior wall thickness; *AVMG*, aortic valve mean gradient; *PASP*, pulmonary artery systolic pressure; *EOAi*, effective orifice area indexed; *BVD*, bioprosthetic valve dysfunction.

∗ Defined according to the Valve Academic Research Consortium-3 definition as follows: indexed effective orifice area (cm2/m2) for BMI <30: moderate = 0.85-0.66 cm2/m2 and severe = ≤ 0.65 cm2/m2 and indexed effective orifice area (cm2/m2) for BMI ≥ 30: moderate = 0.70-0.56 cm2/m2 and severe = ≤ 0.55 cm2/m2.

**Table E3:** Postimplant eccentricity and other prespecified multidetector computed tomography measurements: No subclinical leaflet thrombosis versus subclinical leaflet thrombosis

	Overall cohort N = 118	Native valve type		*P* value
		No SLT N = 96	SLT N = 22	
Eccentricity, mean ±*SD*	0.38 (0.08)	0.37 (0.08)	0.39 (0.07)	.329
Frame inflow	0.50 (0.14)	0.49 (0.15)	0.49 (0.11)	.828
Native annulus	0.51 (0.11)	0.51 (0.11)	0.50 (0.09)	.719
Leaflet inflow	0.47 (0.13)	0.46 (0.13)	0.48 (0.11)	.878
Prosthesis constrained - waist	0.32 (0.15)	0.31 (0.15)	0.37 (0.11)	.110
Leaflet outflow	0.22 (0.12)	0.22 (0.12)	0.25 (0.15)	.263
Frame outflow	0.22 (0.14)	0.22 (0.13)	0.26 (0.18)	.263
Implantation depth, mm, mean ± SD	5.92 (3.13)	6.04 (3.21)	5.36 (2.55)	.406
Commissural misalignment, n (%)∗	24 (20.3)	22 (22.9)	2 (9)	.107
Valve to coronary length, mm, mean ±*SD*				
Left coronary	5.94 (1.91)	5.85 (1.90)	6.44 (2.34)	.215
Right coronary	5.47 (2.10)	5.61 (2.11)	5.33 (2.05)	.767

Values are reported as mean and SD, or number and percentage (%). *SLT,* Subclinical leaflet thrombosis.

∗ Defined as at least moderate and an angle ≥ 30° signified an empirically set threshold for significant commissural misalignment.

**Table E4:** Antithrombotic regimen

Antithrombotic regimen	SLT group (n = 22)	No SLT group (n = 96)	SLT group (%)	No SLT group (%)	*P* value
SAPT	18	77	81.8	80.2	
DOAC	3	11	13.6	11.5	
VKA/OAC	1	8	4.5	8.3	
*P* value					.813

*DOAC,* Direct oral anticoagulation; *OAC,* oral anticoagulation; *SAPT,* single antiplatelet therapy; *SLT,* subclinical leaflet thrombosis; *VKA,* vitamin K antagonist.

**Table E5:** SHapley Additive exPlanations feature importance

Feature	Mean SHAP value
Bicuspid valve	0.420
Hb baseline	0.160
ΔHCT	0.110
ΔHb	0.100
Platelet nadir	0.090
INR nadir	0.080
PASP baseline	0.079
Leaflet inflow eccentricity	0.078
Max gradient baseline	0.077
ΔRBC	0.075
Serum creatinine	0.070
Mean aPTT	0.069
EOAi	0.065
PASP follow-up	0.064
Mean RBC	0.050
Mean hB	0.049
Inflow eccentricity	0.048
Native annulus eccentricity	0.047
EDD baseline	0.046
White cell count baseline	0.045
Mean PLT	0.044
Overall eccentricity	0.043
ΔWCC	0.040
ΔPLT	0.039
Lymphocyte baseline	0.038
WCC nadir	0.037
VTC right	0.030
Monocyte nadir	0.029
ΔINR	0.027
RBC nadir	0.025
CAD	0.024
Lymphocyte mean	0.023
IVS baseline	0.020
HCT baseline	0.019
Monocyte mean	0.015
Lymphocyte nadir	0.014
VTC left	0.011

*SHAP,* SHapley Additive exPlanations; *HCT,* hematocrit; *hB,* hemoglobin; *INR,* international normalized ratio; *PASP,* pulmonary artery systolic pressure; *RBC,* red blood cell; *aPTT,* Activated partial thromboplastin time; *EOAi,* indexed effective orifice area; *EDD,* end-diastolic diameter; *WCC,* white cell count; *PLT,* platelet; *VTC,* valve-to-coronary; *CAD,* coronary artery disease; *IVS,* interventricular septum.

## Supplementary Data

Video 1 A case of severe subclinical leaflet thrombosis visualized using advanced multiparametric digital analysis. The video highlights moderate restriction of leaflet motion. Video available at: https://www.jtcvs.org/article/S2950-6050(25)00023-3/fulltext.

## Appendix E1 Study Population: Inclusion and Exclusion Criteria

Patients with severe symptomatic aortic stenosis with indication for TAVR (transfemoral route).

Inclusion criteria:

All patients with severe symptomatic aortic stenosis with Evolut R/PRO, Portico/Navitor implants.

Exclusion criteria.

i. Aneurysm of the Valsalva or ascending aorta
ii. Previous root replacement
iii. Pure aortic regurgitation
iv. Other significant valve disease
v. Moderate to severe renal failure (glomerular filtration rate <40)
vi. Impossibility to keep the heart rate below 60 bpm at the time of MDCT scan (ie, poorly controlled atrial fibrillation)
vii. Allergy to iodine contrast and other contraindication to MDCT
viii. Patients unwilling to participate in the study or incapable of signing consent form or participating in another study

The use of dual antiplatelet therapy, vitamin K antagonists, or new oral anticoagulants does not represent exclusion criteria.

MDCT Protocol and Analysis.

- Image acquisition.

Contrast-enhanced MDCT was performed using Siemens Healthcare GmbH SOMATOM Drive (VB 20, 2019).

An antecubital intravenous access site was placed (at least a 20-gauge cannula) to deliver iodinated contrast (50-100 mL). Electrocardiogram-gated synchronized imaging was performed to achieve full cardiac cycle acquisition. Image reconstruction was performed in 5% to 10% increments of the R-R interval (equating to 10-20 separate phases). Slice thickness was set to 0.5 mm to improve diagnosis accuracy. The standard tube potential for the procedure was maintained between 100 and 120 kV or higher for obese patients (140 kV) or for patients with other intracardiac devices.

Care was taken to perform image acquisition while maintaining a heart rate below 70 bpm because a higher rate may result in image artefacts. Poorly controlled atrial fibrillation represented an exclusion criterion. Beta-blockers (intravenously) could be used to reduce the heart rate. Images were acquired during peak arterial enhancement in the ascending aorta. This was performed during contrast bolus tracking with automatic triggers once the aortic enhancement reaches more than 300 Hounsfield units.
