# Supplementary figures and images for: Predicting subclinical leaflet thrombosis in self-expandable prosthesis: A multimodal machine learning analysis
Source: JTCVS Struct Endovasc. 2025 Jul 29;8:100064. doi: 10.1016/j.xjse.2025.100064 (PMC13244739; doi:10.1016/j.xjse.2025.100064)

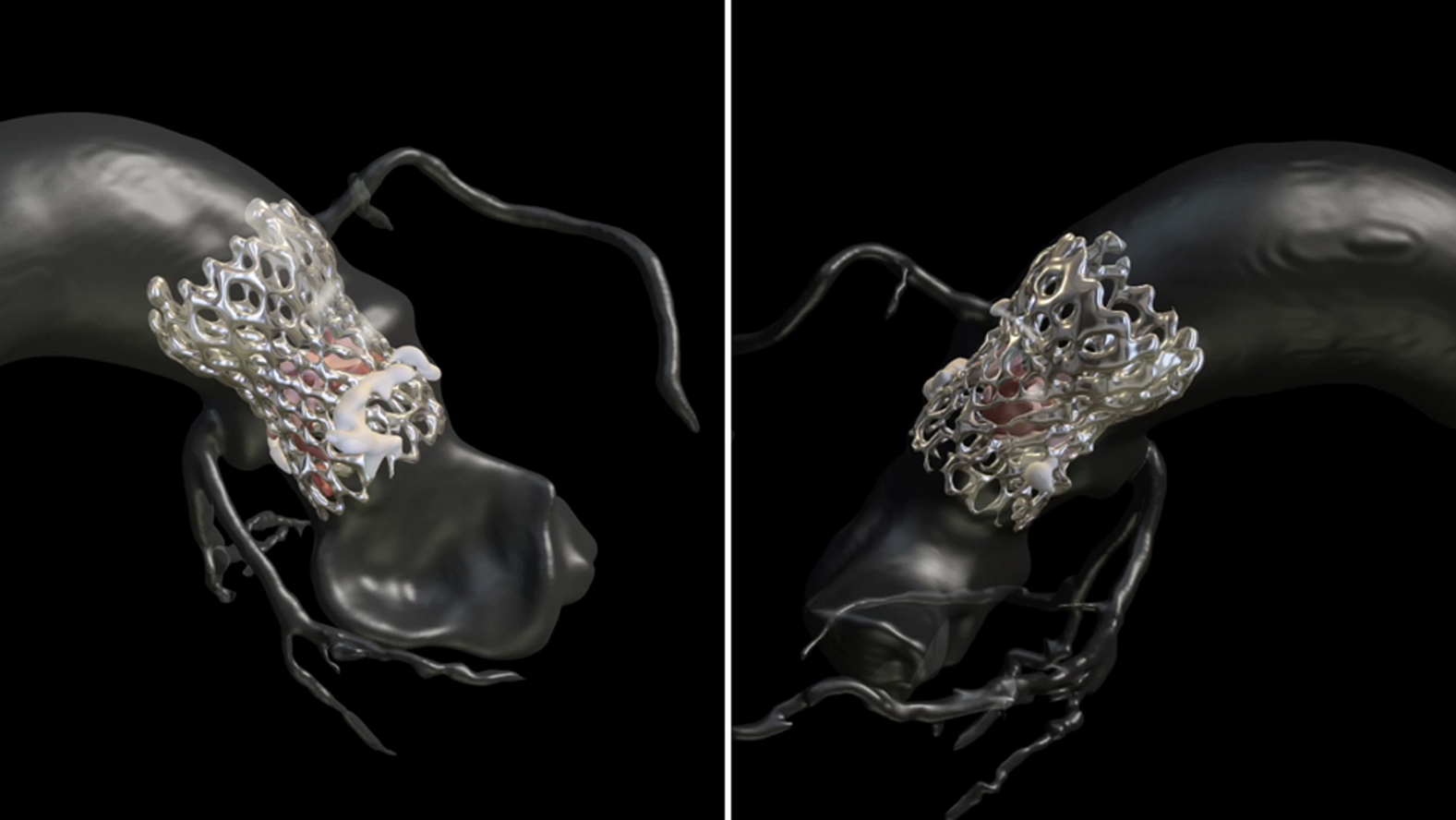

Supplement: Video 1 — A case of severe subclinical leaflet thrombosis visualized using advanced multiparametric digital analysis. The video highlights moderate restriction of leaflet motion. Video available at: https://www.jtcvs.org/article/S2950-6050(25)00023-3/fulltext. [file fx2.jpg]
